# A Structured Narrative Review of the OSA–T2DM Axis

**DOI:** 10.3390/jcm14124168

**Published:** 2025-06-12

**Authors:** Desiderio Passali, Luisa Maria Bellussi, Mariaconsiglia Santantonio, Giulio Cesare Passali

**Affiliations:** 1 ENT Clinic, University of Siena, 53100 Seville, Spain; d.passali@virgilio.it (D.P.); l.bellussi@virgilio.it (L.M.B.); 2 Complex Operational Unit of Ear, Nose and Throat Sciences, Fondazione Policlinico Universitario A. Gemelli, Istituto di Ricovero e Cura a Carattere Scientifico (IRCCS), 00168 Rome, Italy; giuliocesare.passali@unicatt.it

**Keywords:** OSAS, T2DM, diabetes mellitus, sleep disorders, obstructive sleep apnea

## Abstract

**Background/Objectives**: Obstructive sleep apnea (OSA) and type 2 diabetes mellitus (T2DM) are two highly prevalent and interconnected conditions with significant implications for morbidity and mortality. Emerging evidence suggests a bidirectional relationship between the two disorders, mediated by shared pathophysiological mechanisms such as intermittent hypoxia, systemic inflammation, and autonomic dysfunction. **Methods**: A structured narrative review of the literature was conducted using a comprehensive PubMed search of clinical and observational studies published between 2020 and 2024. Studies evaluating the association between OSA and diabetes, including its effects on glycemic control, diabetic complications, and treatment outcomes, were included. **Results**: Thirty-three studies met our inclusion criteria. OSA is independently associated with impaired glucose metabolism, increased insulin resistance, and a higher risk of diabetic complications, including nephropathy, retinopathy, and neuropathy. Continuous positive airway pressure (CPAP) therapy has shown variable effects on metabolic outcomes, largely dependent on adherence. Traditional OSA severity metrics, such as the apnea–hypopnea index (AHI), did not consistently predict metabolic burden. Factors such as sleep quality, nocturnal hypoxemia, and comorbid insomnia have emerged as the most relevant predictors. Sex-specific differences and the roles of pharmacological and behavioral interventions were also noted. **Conclusions**: OSA is a modifiable and under-recognized risk factor for poor glycemic control and diabetes complications. Routine screening and individualized treatment strategies are warranted, particularly for patients with T2DM and suboptimal metabolic control. Future research should focus on defining the phenotypes at the greatest risk and developing integrated treatment pathways.

## 1. Introduction

### 1.1. Diabetes Mellitus

Diabetes mellitus is a chronic and multifaceted metabolic disease that affects hundreds of millions of individuals worldwide. Globally, it is estimated that approximately 463 million adults aged 20–79 years were living with diabetes in 2019, a number projected to reach 700 million by 2045, largely due to aging populations, lifestyle factors, and improved survival rates among diabetic individuals [1].

Type 2 diabetes mellitus (T2DM) accounts for the vast majority of cases and is primarily characterized by insulin resistance and relative insulin deficiency. Although obesity and metabolic syndrome are well-established risk factors, recent research has demonstrated that a substantial proportion of T2DM patients (up to 45%) may present without any clinical features of metabolic syndrome, suggesting distinct underlying pathophysiological mechanisms, such as isolated beta-cell dysfunction [2]. This heterogeneity complicates the diagnosis and management of the disease.

Moreover, the triglyceride–glucose (TyG) index, a surrogate marker of insulin resistance, has been shown to be strongly associated with adverse cardiovascular outcomes in diabetic populations, further underlining the systemic burden of the disease [3].

On the other hand, type 1 diabetes mellitus (T1DM) is an autoimmune condition resulting in the progressive destruction of pancreatic β-cells, leading to absolute insulin deficiency [4,5,6]. It typically manifests during childhood or adolescence but can also occur in adults. The incidence of T1DM has been rising by 2–5% annually, particularly among children under five years of age, indicating potential environmental triggers acting on genetically susceptible individuals.

Although T1DM is briefly introduced, none of the studies included in this review specifically addressed the relationship between obstructive sleep apnea (OSA), obstructive sleep apnea syndrome (OSAS), and T1DM. Therefore, the analysis and conclusions focused exclusively on T2DM and related metabolic conditions.

### 1.2. Sleep Disorders—OSAS

Sleep disorders encompass a heterogeneous group of conditions that disrupt the quantity, quality, or timing of sleep, with implications for overall health, cognitive performance, and metabolic regulation [7,8]. Among sleep disorders, obstructive sleep apnea is both common and increasingly recognized as a contributor to poor health outcomes, including metabolic disturbances related to diabetes.

OSAS is characterized by recurrent episodes of partial or complete upper airway obstruction during sleep, leading to intermittent hypoxia (IH), sleep fragmentation, sympathetic nervous system activation, and increased oxidative stress [9,10,11]. Its prevalence is increasing worldwide, largely driven by rising rates of obesity and aging populations, with estimates indicating that up to 24% of men and 9% of women in the general population aged 30–60 years may be affected [12,13]. Despite its high prevalence, OSAS remains significantly underdiagnosed, often due to nonspecific symptoms and limited access to specialized testing.

The pathophysiological consequences of OSAS are numerous. Recurrent hypoxia and arousal lead to a cascade of events, including

- oxidative stress,
- systemic inflammation,
- endothelial dysfunction,
- and hormonal dysregulation.

These processes have been implicated in the pathogenesis of various chronic diseases, notably cardiovascular, neurological, and metabolic disorders, including insulin resistance and T2DM.

OSAS and metabolic disease appear to have bidirectional influences. On the one hand, sleep fragmentation and nocturnal hypoxemia contribute to impaired glucose metabolism and glycemic variability. On the other, metabolic dysregulation and obesity exacerbate upper airway collapsibility and compromise ventilatory control during sleep. These findings suggest that OSAS is not only a comorbidity but is also potentially an active contributor to the onset and progression of diabetes, especially T2DM.

The diagnosis of OSAS relies on polysomnography or validated home sleep testing tools, with the apnea-hypopnea index (AHI) serving as the standard severity metric. Treatment strategies include continuous positive airway pressure (CPAP) therapy, mandibular advancement devices, lifestyle modifications, and maxillomandibular advancement surgery in selected cases, especially when anatomical factors are predominant [14,15,16].

As evidence continues to mount regarding the close interconnection between OSAS and metabolic health, particularly diabetes [17,18], the need to clarify the mechanisms and clinical implications of this relationship has become increasingly compelling—especially from a multidisciplinary perspective that includes otolaryngology.

Throughout this review, the terms “OSA” (obstructive sleep apnea) and “OSAS” (obstructive sleep apnea syndrome) are used interchangeably, depending on the terminology employed in the original studies. While “OSA” is now the more commonly adopted term in international literature, “OSAS” is sometimes used to specifically refer to cases of obstructive sleep apnea associated with clinically significant daytime symptoms, such as excessive sleepiness. For consistency with the source material, both terms appear in the text where appropriate.

## 2. Materials and Methods

### 2.1. Research and Screening of Literature

This review follows a structured narrative format, combining systematic search strategies with qualitative synthesis and clinical interpretation. A comprehensive literature search was conducted using the PubMed database to identify studies investigating the association between OSA/OSAS and diabetes. The following Boolean search string was used:

*(“obstructive sleep apnea” [MeSH Terms] OR “obstructive sleep apnea” OR “OSA” OR “OSAS” OR “sleep-disordered breathing”) AND (“diabetes mellitus” [MeSH Terms] OR “type 2 diabetes” OR “type 1 diabetes” OR “prediabetes” OR “glucose intolerance” OR “insulin resistance”).*

**Figure 1:**
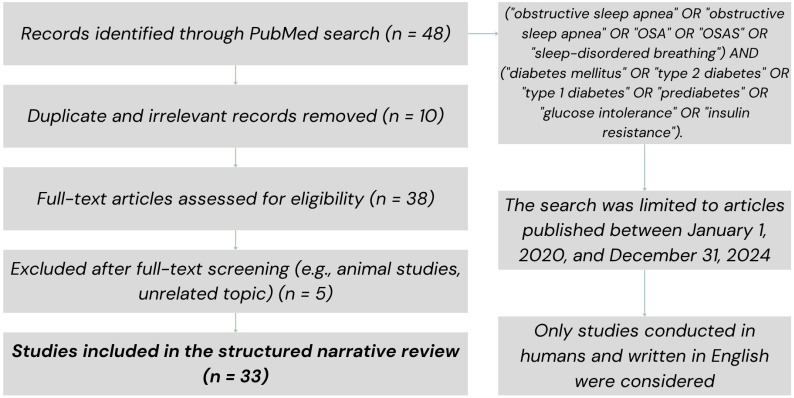
Flow diagram of the literature selection process. A total of 48 articles were retrieved through a database search. After removing duplicates and screening the titles and abstracts, 33 studies were included in the final analysis.

The search was limited to articles published between 1 January 2020, and 31 December 2024. A diagram of the literature selection process is shown in Figure 1.

Central sleep apnea (CSA) was not included in this review. Our search strategy and inclusion criteria were specifically designed to focus on obstructive sleep apnea (OSA/OSAS), which presents distinct pathophysiological mechanisms and clinical relevance compared with CSA.

Only studies conducted in humans written in English were considered. Additional filters were applied to include specific study types, namely, clinical trials, observational studies, comparative studies, classical articles, and clinical studies while excluding preprints. Titles and abstracts were screened for relevance, and the full texts of the selected articles were reviewed for eligibility. Studies that did not directly address the association between OSAS and diabetes were excluded. Additional relevant publications were identified by manual reference checks.

The time window of 2020–2024 was deliberately chosen to reflect the most recent advances in the field and capture emerging insights into the OSA–T2DM relationship. In recent years, the literature has increasingly emphasized novel diagnostic markers beyond AHI, the metabolic impact of comorbid conditions such as insomnia, and the introduction of new pharmacological therapies (e.g., SGLT2 inhibitors) that influence both glycemic control and sleep parameters. This time restriction ensures that the review focuses on up-to-date evidence and evolving clinical paradigms relevant to the current practice.

Of the 48 articles initially retrieved, duplicates and irrelevant studies were removed after independent screening by two reviewers. Discrepancies were resolved through discussion or consultation with a third reviewer when needed. The remaining studies were assessed through full-text analysis, leading to the inclusion of 33 studies in the review.

### 2.2. Inclusion and Exclusion Criteria

The inclusion criteria for this review were as follows: studies published from 2020 onward, indexed in PubMed or other relevant databases, and studies specifically investigating the association between sleep disorders and diabetes.

The exclusion criteria included duplicate publications, articles not directly relevant to the topic (excluded after screening of titles and abstracts), and studies focusing exclusively on animal models.

## 3. Results

### 3.1. Overview

A comprehensive analysis of the literature reveals a growing body of evidence linking OSA and T2DM across a wide spectrum of clinical and research settings. The studies included in this review demonstrate considerable heterogeneity in terms of design, population characteristics, and sample size—with cohorts ranging from fewer than 20 participants [19,20] to large-scale datasets encompassing over 20,000 individuals [21,22,23]. This variation reflects the complexity of investigating the OSA-diabetes interplay, which spans the mechanistic, observational [24,25], and interventional domains [26,27,28].

Collectively, the evidence underscores a bidirectional relationship: OSA contributes to metabolic dysfunction and is influenced by diabetic comorbidities [29,30]. Several studies have highlighted that OSA is independently associated with poorer glycemic control [31,32], increased insulin resistance [24,33], and an elevated risk of diabetic complications, including nephropathy [22,25], retinopathy [21,34], and neuropathy [28,35].

A 2021 study demonstrated a significant association between OSA severity and early peripheral atherosclerosis in hospitalized T2DM patients [36]. Using the ankle-brachial index (ABI) and brachial-ankle pulse wave velocity (PWV) as vascular markers, the study found that higher AHI scores were independently associated with worse vascular profiles and an increased prevalence of lower extremity arterial disease, supporting OSA as a contributor to macrovascular diabetic complications.

In addition, therapeutic interventions such as continuous positive airway pressure (CPAP) have shown mixed results; some studies report modest metabolic benefits, particularly in patients with high adherence [26,27,37], while others suggest a limited impact on glycemic outcomes [38,39,40].

A subset of studies explored novel markers (e.g., cathepsin S [30] and mitochondrial function [20]) and pharmacological agents (e.g., SGLT2 inhibitors [23,41] and metformin [20]), offering new insights into the pathophysiological mechanisms linking these two conditions. The role of lifestyle interventions and comorbid insomnia has also emerged as a relevant factor [42,43], with insomnia sometimes exerting a greater metabolic burden than OSA alone [29,31].

Overall, the reviewed studies reinforce the importance of recognizing and addressing OSA in patients with T2DM, not only because of its impact on metabolic control but also because of its potential role in the development of complications. At the same time, they pointed to the need for more personalized treatment approaches and longer-term studies to better understand how—and for whom—these interventions work best.

### 3.2. Additional Observations and Considerations

***Heterogeneity in Study Designs and Populations***

The reviewed studies encompassed a wide array of populations, including general adults with T2DM, hospitalized patients, those with diabetic complications (e.g., retinopathy, neuropathy, kidney disease), prediabetic individuals, and pregnant women. This heterogeneity underscores the need for a stratified analysis.

For instance, the impact of OSA may differ between early-stage diabetes and long-standing or insulin-treated cases [34,44,45].

***Inconsistency in Diagnostic and Outcome Measures***

Not all studies used the same criteria to diagnose OSA (e.g., AHI thresholds, in-lab vs. home studies) or the same metabolic endpoints (HbA1c, CGM, insulin sensitivity, etc.), which complicates direct comparison and meta-analysis. Some rely on self-reported sleep disorders [29], while others use rigorous polysomnography or genotyping [22].

***Limitations of AHI and Glycemic Assessment Tools in OSA–T2DM Patients***

While AHI remains the gold standard for classifying OSA severity, emerging evidence suggests that AHI alone may not fully capture the metabolic burden of sleep-disordered breathing, particularly in individuals with T2DM. Several studies included in this review point toward a disconnect between AHI-defined severity and metabolic impact, raising questions about the adequacy of traditional OSA classifications in this context.

For example, in a study published in 2020 [42] involving 145 adults with T2DM, OSA severity was not significantly associated with mood, diabetes-related distress, or daytime functioning, whereas comorbid insomnia severity emerged as a powerful predictor of psychological and functional outcomes. Similarly, a more recent study from 2023 [31] confirmed that insomnia severity moderated the relationship between OSA and mood but not diabetes distress, reinforcing the idea that other sleep disturbances may exert a stronger metabolic and emotional toll than OSA.

Patients with insomnia alone had significantly worse glycemic control (higher HbA1c) than those with OSA alone, even though the latter group had a higher BMI [29]. This paradox suggests that sleep fragmentation and poor sleep quality, regardless of AHI, may play a more prominent role in glucose dysregulation than was previously thought.

Individuals with moderate-to-severe OSA exhibit peripheral insulin resistance in the skeletal muscle and adipose tissue, even though hepatic insulin sensitivity remains unaffected [24].

This suggests that AHI does not capture all the relevant metabolic consequences, particularly those related to tissue-specific insulin action.

Collectively, these findings challenge the primacy of AHI as the sole marker for OSA severity in the diabetic population. They called for a broader conceptual framework that includes the following:

- oxygen desaturation burden (e.g., ODI),
- sleep architecture parameters (e.g., arousal index, REM sleep percentage),
- and comorbid conditions like insomnia and depression, all of which may influence metabolic risk.

In parallel, a study published in 2023 [46] highlighted that even metabolic assessment tools commonly used in this population may present limitations. In their study involving T2DM patients with untreated OSA, they observed marked discrepancies between the Glucose Management Indicator (GMI) derived from continuous glucose monitoring and laboratory-measured HbA1c levels. This raises concerns about overreliance on CGM-derived estimates in patients with sleep-disordered breathing and suggests the need for multimodal metabolic evaluation approaches.

***Sex Differences and Subgroup Effects***

Some findings indicate that the effect of OSA or its treatment differs by sex—e.g., positive air pressure (PAP) therapy significantly improved glucose variability in women but not in men [26]. This supports the need for sex-specific analyses in future research and personalized medical approaches.

***OSA as a Modifiable Risk Factor for Diabetic Complications***

Several studies suggest that OSA not only worsens glycemic control but also contributes to the progression of diabetes-related complications—retinopathy [21], nephropathy [25], cardiovascular events [44], and stroke [47]. This finding supports the concept that OSA screening should be integrated into diabetes care pathways, especially in patients with complications.

***The Challenge of CPAP Adherence***

One of the most critical factors influencing the efficacy of CPAP therapy in patients with T2DM and OSA is adherence [48]. Multiple studies have demonstrated that suboptimal use of CPAP—typically defined as fewer than 4 h per night—may blunt or eliminate its potential metabolic benefits. This issue is especially relevant in diabetic populations, where comorbid conditions, reduced sleep quality, and psychological distress may further compromise adherence.

The Diabetes Sleep Treatment Trial [27] reported no significant improvement in HbA1c in the intention-to-treat analysis; however, they found a clear dose-response relationship between CPAP usage and glycemic improvements, and only participants using CPAP for ≥7 h/night showed meaningful HbA1c reductions. Similarly, in another study [26], glycemic variability was reduced only in female patients, with significant effects observed in those with consistent nightly CPAP use.

A feasibility trial [28] also confirmed that median CPAP adherence was low (~3.5 h/night), limiting its clinical impact. The authors suggest that a “run-in” period prior to randomization could improve long-term adherence and trial retention. Poor adherence was also a challenge in the SAVE substudy [39], which failed to show any significant improvement in glycemic outcomes or diabetes prevention with CPAP, possibly due to insufficient usage.

Physical inactivity, frequently observed in individuals at risk of OSA, may exacerbate metabolic and cardiovascular risks. A study published in 2020 [49] found that over one-third of adults awaiting OSA diagnosis did not meet the WHO physical activity guidelines, with low motivation and pain as major barriers. This underlines the need for behavioral and motivational strategies to complement OSA management in patients with diabetes.

In addition to CPAP and pharmacological therapy, bariatric surgery may offer dual benefits to patients with OSA and diabetes. A multicenter study [50] reported that Roux-en-Y gastric bypass was associated with higher remission rates of both OSA (64.5%) and insulin-dependent T2DM than sleeve gastrectomy, suggesting that surgical approach selection is crucial in obese diabetic patients with sleep-disordered breathing.

Further support for the metabolic benefits of CPAP was provided through a randomized controlled trial in prediabetic patients [51]. They demonstrated that optimal CPAP use significantly reduced resting heart rate and plasma norepinephrine levels, reinforcing the role of sympathetic activation in the OSA-diabetes axis and suggesting preventive potential for diabetes progression.

Table 1 provides an overview of the results discussed in this section.

## 4. Discussion

### 4.1. A Complex and Bidirectional Relationship

This narrative review supports the growing consensus that OSA and T2DM are tightly interconnected through complex bidirectional relationships. OSA is highly prevalent among individuals with T2DM and has been consistently linked to poor glycemic control and progression of diabetic complications, including nephropathy, retinopathy, and neuropathy. Conversely, poorly controlled diabetes may worsen OSA through autonomic dysfunction, peripheral neuropathy, and upper airway inflammation.

A key insight from this review is the recognition that traditional measures of OSA severity—particularly the AHI—may fail to capture the full extent of OSA’s metabolic impact. Multiple studies have indicated that markers like nocturnal hypoxemia, sleep fragmentation, and comorbid insomnia better reflect the degree of metabolic dysregulation in T2DM patients. These findings underscore the need for a more nuanced assessment framework that extends beyond the AHI and includes sleep quality and oxygenation parameters.

The pathophysiological pathways through which OSA contributes to the development and progression of T2DM are multifactorial and involve several interrelated mechanisms.

One of the most critical factors is IH, a hallmark of OSA that induces oxidative stress and stimulates the production of pro-inflammatory cytokines [24,26,35]. These processes lead to systemic inflammation, which plays a direct role in the development of insulin resistance and impaired glucose metabolism.

Another key mechanism is the chronic activation of the sympathetic nervous system caused by repeated arousals during sleep. Autonomic overactivation increases hepatic glucose output and disrupts insulin signaling pathways. The metabolic impact of sympathetic overdrive is supported by findings from a randomized trial [51], where the optimal use of CPAP significantly reduced plasma norepinephrine levels and resting heart rate in individuals with prediabetes.

Additionally, OSA is associated with several hormonal alterations that further contribute to metabolic dysregulation. These include elevated levels of cortisol and reduced concentrations of adipokines, both of which negatively affect insulin sensitivity [30,35].

These mechanisms are also linked to endothelial dysfunction, which impairs vascular insulin action and may contribute to both micro- and macrovascular diabetic complications, as demonstrated in vascular studies using PWV and ABI metrics [36].

Importantly, most of the studies included in this review adjusted for obesity-related factors, such as BMI, waist circumference, and VFA, and still observed a significant association between OSA and metabolic dysfunction. This observation suggests that OSA may serve as an independent contributor to glycemic instability and diabetes-related complications rather than acting merely as a mediator of obesity-related effects.

Collectively, these mechanisms offer a biologically plausible and clinically relevant explanation for the consistent association observed between OSA and T2DM in multiple studies. A deeper understanding of these pathways is essential for the identification of high-risk patient profiles and for the development of individualized therapeutic strategies, which may include not only CPAP therapy but also pharmacological and behavioral interventions tailored to address both sleep and metabolic dysfunction.

On the other hand, it is important to highlight that while CPAP therapy remains the standard treatment for OSA, its metabolic benefits appear to be strongly influenced by patient adherence. Studies have shown that only patients with consistent nightly use, typically ≥7 h, experience meaningful improvements in glycemic outcomes. This underlines the importance of behavioral interventions, patient education, and adherence monitoring as essential components of OSA treatment in diabetic populations. The reviewed literature is heterogeneous in design, population, and outcome metrics, ranging from mechanistic studies to randomized controlled trials. While this reflects the multifactorial nature of the OSA–diabetes interplay, it also limits comparability across studies and underscores the need for standardized research protocols.

**Figure 2:**
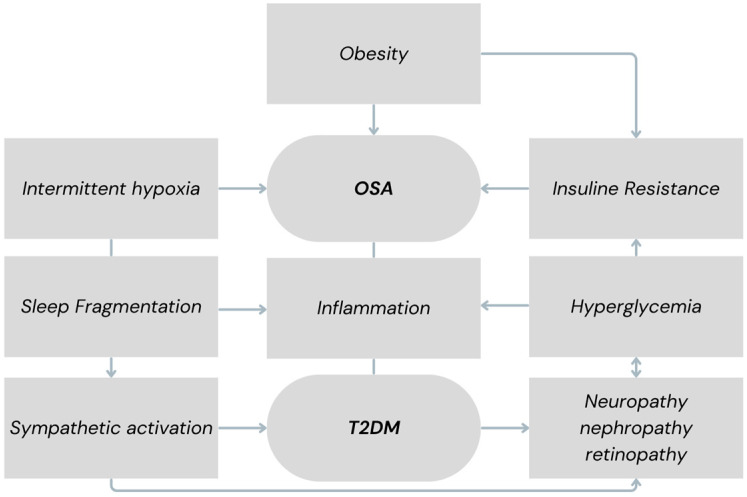
Schematic representation of the bidirectional relationship between OSA and T2DM. Key mediators include obesity, intermittent hypoxia, sympathetic activation, systemic inflammation, and insulin resistance. Note: This diagram refers exclusively to obstructive sleep apnea (OSA/OSAS).

The main elements presented in the results are summarized in Table 2 for clarity and are visually represented in Figure 2.

### 4.2. Clinical Implications and Future Directions

Given the consistent association between OSA and adverse diabetic outcomes, routine screening for OSA in individuals with T2DM, especially in those with complications or poor glycemic control, should be strongly considered. Treatment strategies must also be individualized, with particular attention paid to patient-reported symptoms, sleep quality, and behavioral barriers to CPAP adherence. Future research should focus on the following aspects:

- defining OSA phenotypes most at risk for metabolic complications,
- developing multimodal interventions combining CPAP, pharmacotherapy, and behavioral therapy,
- and identifying robust biomarkers that can guide therapy and predict outcomes.

On the other hand, obesity is a key determinant of both OSA and T2DM, contributing to upper airway collapsibility, insulin resistance, and systemic inflammation. Several studies included in this review accounted for obesity in their analyses, either through stratified models or by adjusting for BMI, and demonstrated that the association between OSA and T2DM often persists independently of adiposity.

However, obesity may exacerbate the impact of OSA on diabetes-related complications, such as nephropathy and retinopathy, underscoring the importance of integrated management strategies in this subgroup of patients.

Importantly, the metabolic burden of OSA was observed even in non-obese individuals, suggesting that OSA and T2DM are also linked through weight-independent mechanisms, such as intermittent hypoxemia, oxidative stress, and sympathetic overactivation. These findings reinforce the need for routine OSA screening in all T2DM patients, regardless of body weight.

This review offers a contribution by not only highlighting the bidirectional relationship between OSA and T2DM but also by emphasizing the need to move beyond traditional diagnostic metrics such as the AHI, which may inadequately capture true metabolic risk in diabetic patients. The strength of this work lies in its synthesis of recent heterogeneous studies, allowing for the identification of emerging clinical patterns and critical knowledge gaps.

### 4.3. Limitations of the Reviewed Literature

However, it is important to note the limitations of the existing body of evidence. Many studies rely on cross-sectional designs, limiting causal inferences. Others include small sample sizes or a lack of long-term follow-up. Moreover, adherence to CPAP and other interventions has been inconsistently reported or is suboptimal. These issues should be addressed in future trials through a better study design, longer observation periods, and improved patient engagement strategies. Furthermore, the majority of the included studies were observational in nature, often with heterogeneous designs and variable sample sizes. While we did not perform a formal risk of bias assessment, we acknowledge the potential for selection and publication bias and have highlighted methodological limitations when applicable throughout the discussion.

**Figure 3:**
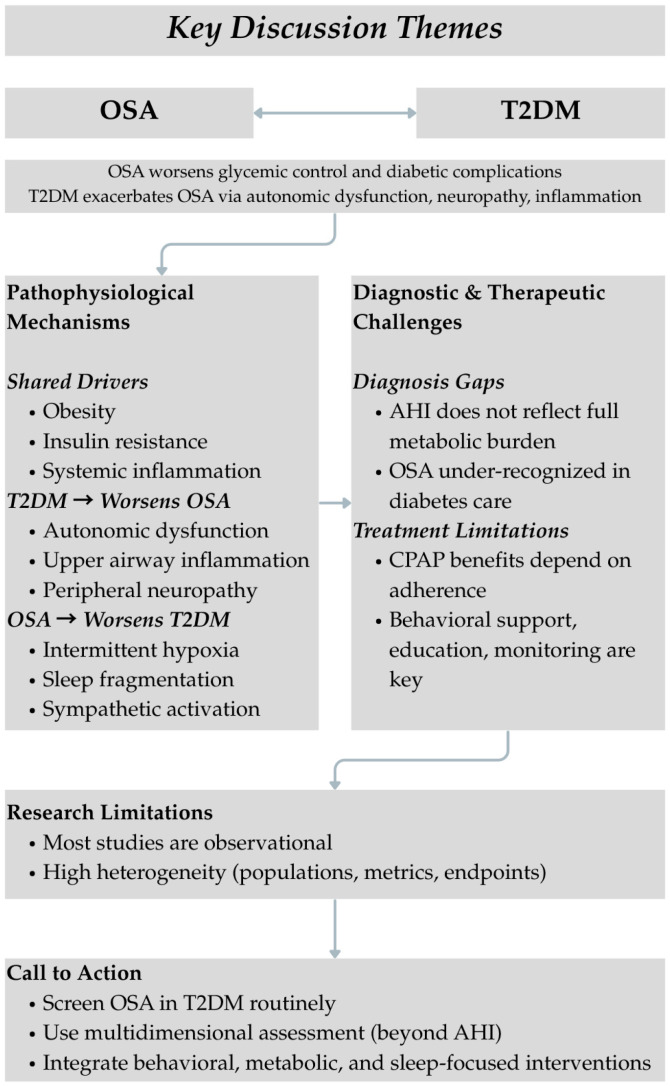
Summary of Key Discussion Themes: The OSA–T2DM Interplay.

A visual overview of this discussion is presented in Figure 3.

## 5. Conclusions

The evidence reviewed in this narrative analysis strongly supports a clinically significant association between OSA/OSAS and T2DM, with implications that extend beyond glycemic control to include the risks of chronic complications and mortality. While OSA is prevalent among individuals with T2DM, its diagnosis and treatment remain under-recognized in routine diabetes care.

AHI-defined OSA severity does not reliably predict metabolic outcomes, suggesting the need for broader assessments, including sleep quality and comorbidities like insomnia. CPAP can improve glycemic control in adherent users; however, poor compliance limits its impact. Emerging pharmacological, behavioral, and surgical options may complement the standard therapies. Given its role in worsening diabetes, OSA should be routinely screened for in T2DM care, particularly in patients with poor control or complications.

In this narrative review, we aimed to summarize recent findings on the interplay between OSA and T2DM, highlighting clinical observations that may not yet be fully reflected in current diabetes care. While not exhaustive, our overview brings together key studies that support the idea that OSA is a modifiable factor in the metabolic trajectory of many patients. As awareness grows around the OSA–T2DM interplay, incorporating sleep assessment into routine diabetes care may become a standard, low-cost, and high-yield strategy to reduce long-term complications.

## Figures and Tables

**Table 1:** Overview of the results.

Author (Year)	N. Subjects	Category	Main Results	Analyses Adjusted for BMI	Effect of Obesity on OSA–T2DM Association
**Jeon et al. (2020)** [42]	145	Adults with T2DM and, comorbid OSA & insomnia	OSA severity not associated with mood/distress; insomnia more impactful than OSA	BMI was included in the analyses and adjusted for regression models.	OSA was independently associated with higher HbA1c and insulin resistance even after controlling for obesity.
**Rahimy et al. (2025)** [21]	23,862	NPDR patients with/without OSA	OSA is associated with an increased risk of DR progression, systemic complications, and death	Propensity score matching was performed to control for baseline demographics and comorbidities.	The study did not examine the modifying effect of obesity on the OSA–T2DM relationship.
**Wang et al. (2025)** [22]	7980	Adults ≥20 years from NHANES	OSA genetically linked to T2DM; obesity mediates OSA-CKD relationship	The study adjusted for BMI and VFA in all regression models.	The association between OSA (especially severe OSA) and increased insulin resistance remained significant after adjusting for BMI and VFA, indicating an independent contribution of OSA, although obesity also played a role.
**Li et al. (2021)** [32]	11,623	USA Hispanic/Latino adults	SDB independently associated with incident T2DM and hypertension	BMI was included as a covariate in multivariable regression analyses.	The association between OSA severity and glycemic variability remained significant after adjusting for BMI, though BMI itself was also significantly associated with glucose outcomes.
**Vichova et al. (2025)** [44]	453	Patients with DM from the SHHS cohort	Severe SDB increases all-cause and cardiovascular mortality in T2DM	The study used adjusted hazard ratios in proportional hazard models	Not analyzed/thoroughly discussed
**Barreto et al. (2020)** [47]	102	Patients with ischemic stroke	T2DM independently associated with wake-up stroke; OSA linked to worse 1-year outcomes	N/A	N/A
**Makhdom et al. (2024)** [28]	83	T2DM and OSA patients	Feasibility trial: CPAP showed possible benefits on CKD, neuropathy, and QoL	N/A	The study observed a potential favorable association between CPAP use and diabetes-related complications (chronic kidney disease and neuropathy), but did not assess how obesity modifies this association.
**Jeon et al. (2023)** [31]	240	Adults with T2DM and OSA	Insomnia moderates the OSA-mood link; insomnia is independently linked to distress.	N/A	N/A
**Zhao et al. (2021)** [36]	312	T2DM patients with habitual snoring	OSA severity associated with early atherosclerosis and LEAD	BMI was measured and included as an adjustment factor in multivariate regression analyses.	The association between OSA and early atherosclerosis (ABI, PWV, and LEAD) remained significant after adjusting for BMI.
**Chasens et al. (2022)** [27]	98	Adults with T2DM and OSA	CPAP use linked to improved HbA1c in adherent users	N/A	N/A
**Banghøj et al. (2020)** [38]	72	T2DM with newly diagnosed OSA	12-week CPAP did not significantly improve glycemic control	Analyses adjusted for BMI and obesity were considered in the interpretation of results.	The study found that the association between OSA and insulin resistance persisted after adjusting for BMI, but BMI remained a relevant factor.
**Krogager et al. (2020)** [40]	72	T2DM and OSA patients	CPAP did not reduce BP or arterial stiffness significantly	N/A	N/A
**Rhodes et al. (2020)** [49]	60	Adults at high risk for OSA	Physical inactivity common in T2DM + OSA; linked to lower motivation	N/A	N/A
**Koh et al. (2023)** [24]	28	Obese, non-diabetic adults with/without OSA	OSA impairs insulin sensitivity in skeletal muscle/adipose tissue	The study adjusted for BMI and other cardiometabolic risk factors in its analyses.	OSA was associated with impaired insulin sensitivity and glycemic variability independent of BMI, suggesting that OSA contributes to T2DM risk beyond the effect of obesity.
**Loffler et al. (2020)** [39]	888	OSA with comorbid CVD	CPAP did not improve glycemic control or reduce diabetes incidence	The study used multivariable Cox regression models adjusted for BMI and other cardiometabolic risk factors.	The bidirectional association between OSA and T2DM remained significant even after adjusting for obesity.
**Villalaín-Rodes et al. (2025)** [34]	83	Patients with NPDR and OSA	CPAP improved AVR and arteriolar diameter, suggesting retinal benefits	N/A	N/A
**Pavlou et al. (2024)** [43]	75	Adults with T2DM	Time-restricted eating improved HbA1c but not OSA risk or sleep quality	All participants were obese at baseline (BMI 30–50 kg/m^2^), and analyses considered BMI-related effects across intervention arms.	Although weight loss was a central intervention, the study did not directly assess how obesity modifies the OSA–T2DM relationship. It notes that a >10% weight reduction might be needed to improve OSA risk, but no causal pathway analysis was done.
**Zunica et al. (2024)** [20]	16	Obese adults on APAP	Metformin preserved mitochondrial function and HbA1c in OSA patients	BMI and waist circumference were recorded and included as covariates in multivariate regression analyses.	The association between higher OSA severity and impaired glycemic variability persisted after adjusting for BMI and waist circumference.
**Aurora et al. (2023)** [26]	184	T2DM and OSA adults	CPAP improved glucose variability in women, not men	Participants were stratified by BMI (<35 vs. ≥35 kg/m^2^), and subgroup analyses were conducted accordingly.	No significant differences in CGM outcomes by BMI group were found, although some SMBG improvements were observed in participants with BMI ≥ 35.
**Wojeck et al. (2023)** [23]	7697	T2DM with ASCVD (without baseline OSA)	Ertugliflozin reduced incident OSA by 48%	The stratified Cox regression model adjusted for baseline BMI (categorized as <35 and ≥35 kg/m^2^), along with other covariates like age, sex, HbA1c, and eGFR.	The study did not specifically examine how obesity modifies the relationship between OSA and T2DM. It
**Thaher et al. (2024)** [50]	2524	Morbidly obese patients post-bariatric surgery	RYGB is more effective than SG in OSAS and IDDM remission	BMI was included as a covariate in the multivariable logistic regression analysis evaluating the association between OSA and T2DM.	The study found that the association between OSA and T2DM remained significant even after adjusting for BMI.
**Briançon-Marjollet et al. (2025)** [19]	9	Healthy non-obese adults	Intermittent hypoxia induces lipid insulin resistance without hyperglycemia.	BMI was matched between OSA and control groups; analyses were adjusted for obesity.	Even with matched BMI, OSA was associated with impaired insulin sensitivity and glycemic variability.
**Imes et al. (2022)** [29]	253	Adults with T2DM and sleep disorders	Insomnia is associated with worse glycemic control than OSA alone	Analyses adjusted for BMI; BMI differences between groups were also explicitly reported and considered.	The group with insomnia alone had higher HbA1c despite lower BMI, suggesting factors beyond obesity influence glycemic control. However, OSA without insomnia was associated with higher BMI but lower HbA1c.
**Moawd et al. (2020)** [35]	55	T2DM with DPN and OSA	Inspiratory muscle training improved aerobic capacity, not glycemia	BMI was measured and reported; the two randomized groups were matched for BMI at baseline.	The study did not specifically analyze how obesity modifies the relationship between OSA and T2DM.
**Zamarrón et al. (2021)** [25]	214	T2DM with diabetic kidney disease	Severe OSA associated with worse renal function in DKD	BMI and waist circumference were measured and adjusted for in the statistical analyses, including multivariable regression models.	The association between OSA severity and poor glycemic control (HbA1c, insulin resistance) remained significant after adjusting for BMI and waist circumference.
**Pamidi et al. (2020)** [51]	39	OSA with prediabetes	CPAP lowered resting heart rate and sympathetic tone	Participants were all overweight or obese (BMI ≥ 25 kg/m^2^), and statistical models adjusted for baseline OSA severity, age, and sex, which are confounders of cardiometabolic outcomes.	All participants had prediabetes and were overweight/obese, but the modifying effect of obesity on the relationship between OSA and glycemic status was not explored independently.
**Fang et al. (2023)** [46]	144	T2DM and untreated OSA	GMI may be unreliable for glycemic assessment in OSA patients	BMI was measured and reported; analyses assessed differences across BMI and other comorbidities.	Results did not significantly differ across BMI or obesity status, but the study did not explicitly focus on this interaction.
**Pinilla et al. (2021)** [33]	599	Adults with suspected OSA	OSA is linked to biological aging and insulin resistance, esp. <50 years	BMI and waist circumference were included as covariates in all statistical models.	OSA was associated with poorer glycemic control (higher HbA1c), and this association remained significant after adjusting for BMI and waist circumference. However, obesity was also independently associated with glycemic metrics.
**Rooney et al. (2021)** [48]	186	T2DM with moderate-to-severe OSA	HYPNOS trial design: assessing CPAP effects on glycemic control	Analyses included BMI as a covariate; comparisons between intervention and control groups accounted for baseline BMI.	Improvements in glycemic control (HbA1c) with CPAP occurred independently of weight loss, suggesting that OSA affects T2DM outcomes beyond the role of obesity.
**Bakker et al. (2020)** [37]	141	T2DM and moderate OSA	CPAP improved vascular function only in adherent users	Analyses adjusted for BMI and waist circumference; BMI was also examined as a moderator.	Higher BMI attenuated the association between OSA and glycemic measures, but OSA remained a significant predictor even after controlling for obesity.
**Neeland et al. (2020)** [41]	7020	T2DM with CVD	Empagliflozin reduced new-onset OSA and improved outcomes	The study performed extensive adjustments for obesity measures, including BMI, waist circumference, and body fat distribution (especially visceral adiposity).	Although visceral adiposity was more strongly associated with insulin resistance and glycemic control than OSA alone, OSA remained independently associated with impaired glucose metabolism after controlling for obesity-related factors.
**Wen et al. (2020)** [30]	158	T2DM with/without OSA	Cathepsin S independently predicts OSA severity in T2DM	BMI was included as a covariate in multivariate Cox proportional hazards models.	The association between OSA and incident T2DM remained significant even after adjusting for BMI and other confounders, suggesting that OSA contributes independently to diabetes risk.

Abbreviations used: AHI = Apnea–Hypopnea Index; CPAP = Continuous Positive Airway Pressure; HbA1c = Hemoglobin A1c; PWV = Pulse Wave Velocity; ABI = Ankle–Brachial Index; CGM = Continuous Glucose Monitoring; ODI = Oxygen Desaturation Index; GMI = Glucose Management Indicator; DKD = Diabetic Kidney Disease; RYGB = Roux-en-Y Gastric Bypass; SG = Sleeve Gastrectomy; ASCVD = Atherosclerotic Cardiovascular Disease; APAP = Automatic Positive Airway Pressure; NPDR = Non-Proliferative Diabetic Retinopathy; VFA = visceral fat area.

**Table 2:** Main finds and themes discussed in this review.

What is Known	What is Unclear	What this Review Adds
OSA is associated with poorer glycemic control and insulin resistance in T2DM	Whether CPAP consistently improves metabolic outcomes in T2DM patients	Summarizes evidence showing that OSA contributes to complications such as neuropathy, nephropathy, and retinopathy
CPAP therapy has shown mixed effects on glycemic outcomes	To what extent does OSA contribute to diabetic complications like nephropathy and retinopathy	Highlights the impact of CPAP adherence, insomnia, and sex differences on treatment outcomes
AHI is commonly used to assess OSA severity	Whether AHI adequately reflects metabolic burden compared to other parameters	Suggests integrating alternative markers (e.g., nocturnal hypoxemia, sleep quality) into OSA assessment in T2DM

## Data Availability

No new data were created.

## Abbreviations

The following abbreviations are used in this manuscript:

ABI	Ankle–Brachial Index
AHI	Apnea–Hypopnea Index
APAP	Automatic Positive Airway Pressure
ASCVD	Atherosclerotic Cardiovascular Disease
BMI	Body Mass Index
CGM	Continuous Glucose Monitoring
CPAP	Continuous Positive Airway Pressure
DKD	Diabetic Kidney Disease
GMI	Glucose Management Indicator
HbA1c	Hemoglobin A1c
IH	Intermittent Hypoxia
NPDR	Non-Proliferative Diabetic Retinopathy
ODI	Oxygen Desaturation Index
OSA	Obstructive Sleep Apnea
OSAS	Obstructive Sleep Apnea Syndrome
PAP	Positive Airway Pressure
PWV	Pulse Wave Velocity
REM	Rapid Eye Movement
RYGB	Roux-en-Y Gastric Bypass
SG	Sleeve Gastrectomy
T1DM	Type 1 Diabetes Mellitus
T2DM	Type 2 Diabetes Mellitus
TyG	Triglyceride–Glucose Index
VFA	Visceral Fat Area

## References

[1] Russo M.P., Grande-Ratti M.F., Burgos M.A., Molaro A.A., Bonella M.B. (2023). Prevalence of diabetes, epidemiological characteristics and vascular complications. Arch. Cardiol. Mexico.

[2] Zhang J., Zhan Q., Deng Z., Lin L., Feng Z., He H., Zhang D., Zhao H., Gu X., Yin X. (2025). Does diabetes modify the triglyceride-glucose index associated with cardiovascular events and mortality? A meta-analysis of 50 cohorts involving 7,239,790 participants. Cardiovasc. Diabetol..

[3] Rottenkolber M., Gar C., Then C., Wanger L., Sacco V., Banning F., Potzel A.L., Kern-Matschilles S., Nevinny-Stickel-Hinzpeter C., Grallert H. (2021). A Pathophysiology of Type 2 Diabetes Unrelated to Metabolic Syndrome. J. Clin. Endocrinol. Metab..

[4] Maahs D.M., West N.A., Lawrence J.M., Mayer-Davis E.J. (2010). Epidemiology of type 1 diabetes. Endocrinol. Metab. Clin. N. Am..

[5] Xia Y., Xie Z., Huang G., Zhou Z. (2019). Incidence and trend of type 1 diabetes and the underlying environmental determinants. Diabetes Metab. Res. Rev..

[6] Bluestone J.A., Herold K., Eisenbarth G. (2010). Genetics, pathogenesis and clinical interventions in type 1 diabetes. Nature.

[7] Jaqua E.E., Hanna M., Labib W., Moore C., Matossian V. (2023). Common Sleep Disorders Affecting Older Adults. Perm. J..

[8] Sun S.Y., Chen G.H. (2022). Treatment of Circadian Rhythm Sleep-Wake Disorders. Curr. Neuropharmacol..

[9] Lavalle S., Masiello E., Iannella G., Magliulo G., Pace A., Lechien J.R., Calvo-Henriquez C., Cocuzza S., Parisi F.M., Favier V. (2024). Unraveling the Complexities of Oxidative Stress and Inflammation Biomarkers in Obstructive Sleep Apnea Syndrome: A Comprehensive Review. Life.

[10] Passali D., Corallo G., Yaremchuk S., Longini M., Proietti F., Passali G.C., Bellussi L. (2015). Oxidative stress in patients with obstructive sleep apnoea syndrome. Acta Otorhinolaryngol. Ital..

[11] Lira A.B., de Sousa Rodrigues C.F. (2016). Evaluation of oxidative stress markers in obstructive sleep apnea syndrome and additional antioxidant therapy: A review article. Sleep Breath..

[12] Faber J., Faber C., Faber A.P. (2019). Obstructive sleep apnea in adults. Dent. Press J. Orthod..

[13] Lv R., Liu X., Zhang Y., Dong N., Wang X., He Y., Yue H., Yin Q. (2023). Pathophysiological mechanisms and therapeutic approaches in obstructive sleep apnea syndrome. Signal Transduct. Target. Ther..

[14] Toraldo D.M., Passali D., Sanna A., De Nuccio F., Conte L., De Benedetto M. (2017). Cost-effectiveness strategies in OSAS management: A short review. Acta Otorhinolaryngol. Ital..

[15] Ruaro B., Baratella E., Confalonieri M., Antonaglia C., Salton F. (2022). Editorial: Obstructive sleep apnea syndrome (OSAS). What’s new?. Front. Med..

[16] Passàli D., Tatti P., Toraldo M., de Benedetto M., Peverini F., Caruso G., Marzetti A., Passàli F.M., Bellussi L. (2014). OSAS and metabolic diseases: Round Table, 99(th) SIO National Congress, Bari 2012. Acta Otorhinolaryngol. Ital..

[17] Tatti P., Strollo F., Passali D. (2013). Sleep apnea, sleep disturbance, and fasting glucose variability: A pilot study. J. Diabetes Sci. Technol..

[18] Tatti P., Tahrani A., Passali D., Reutrakul S., Kanagasabai T. (2019). The Relationship between Disturbed Sleep, OSAS, and Metabolic Diseases. J. Diabetes Res..

[19] Briançon-Marjollet A., Netchitaïlo M., Fabre F., Belaidi E., Arnaud C., Borel A.L., Levy P., Pépin J.L., Tamisier R. (2025). Intermittent hypoxia increases lipid insulin resistance in healthy humans: A randomized crossover trial. J. Sleep Res..

[20] Zunica E.R.M., Heintz E.C., Dantas W.S., Hebert R.C., Tanksley M., Beyl R.A., Mader E.C., Kirwan J.P., Axelrod C.L., Singh P. (2024). Effects of metformin on glucose metabolism and mitochondrial function in patients with obstructive sleep apnea: A pilot randomized trial. Physiol. Rep..

[21] Rahimy E., Koo E.B., Wai K.M., Ludwig C.A., Kossler A.L., Mruthyunjaya P. (2025). Impact of Obstructive Sleep Apnea on Diabetic Retinopathy Progression and Systemic Complications. Am. J. Ophthalmol..

[22] Wang S., Cheng Y., Zhang Z., Liu W., Ou M., Yin T., Meng Y., Ban H., Gu W., Meng X. (2025). Association between obstructive sleep apnea and chronic kidney disease: A cross-sectional and Mendelian randomization study. Medicine.

[23] Wojeck B.S., Inzucchi S.E., Neeland I.J., Mancuso J.P., Frederich R., Masiukiewicz U., Cater N.B., McGuire D.K., Cannon C.P., Yaggi H.K. (2023). Ertugliflozin and incident obstructive sleep apnea: An analysis from the VERTIS CV trial. Sleep Breath..

[24] Koh H.E., van Vliet S., Cao C., Patterson B.W., Reeds D.N., Laforest R., Gropler R.J., Ju Y.S., Mittendorfer B. (2022). Effect of obstructive sleep apnea on glucose metabolism. Eur. J. Endocrinol..

[25] Zamarrón E., Jaureguizar A., García-Sánchez A., Díaz-Cambriles T., Alonso-Fernández A., Lores V., Mediano O., Rodríguez-Rodríguez P., Cabello-Pelegrín S., Morales-Ruíz E. (2021). Obstructive sleep apnea is associated with impaired renal function in patients with diabetic kidney disease. Sci. Rep..

[26] Aurora R.N., Rooney M.R., Wang D., Selvin E., Punjabi N.M. (2023). Effects of Positive Airway Pressure Therapy on Glycemic Variability in Patients with Type 2 Diabetes and OSA: A Randomized Controlled Trial. Chest.

[27] Chasens E.R., Korytkowski M., Burke L.E., Strollo P.J., Stansbury R., Bizhanova Z., Atwood C.W., Sereika S.M. (2022). Effect of Treatment of OSA With CPAP on Glycemic Control in Adults with Type 2 Diabetes: The Diabetes Sleep Treatment Trial (DSTT). Endocr. Pract..

[28] Makhdom E.A., Maher A., Ottridge R., Nicholls M., Ali A., Cooper B.G., Ajjan R.A., Bellary S., Hanif W., Hanna F. (2024). The impact of obstructive sleep apnea treatment on microvascular complications in patients with type 2 diabetes: A feasibility randomized controlled trial. J. Clin. Sleep Med..

[29] Imes C.C., Bizhanova Z., Sereika S.M., Korytkowski M.T., Atwood C.W., Burke L.E., Kariuki J., Morris J.L., Stansbury R., Strollo P.J. (2022). Metabolic outcomes in adults with type 2 diabetes and sleep disorders. Sleep Breath..

[30] Wen W., Sun H., Yang Y., Jia Y., Fang F., Qin Y., Zhang M., Wei Y. (2020). Usefulness of Cathepsin S to Predict Risk for Obstructive Sleep Apnea among Patients with Type 2 Diabetes. Dis. Markers.

[31] Jeon B., Chasens E.R., Luyster F.S., Callan J.A., DiNardo M.M., Sereika S.M. (2023). Is insomnia severity a moderator of the associations between obstructive sleep apnea severity with mood and diabetes-related distress?. Sleep Breath..

[32] Li X., Sotres-Alvarez D., Gallo L.C., Ramos A.R., Aviles-Santa L., Perreira K.M., Isasi C.R., Zee P.C., Savin K.L., Schneiderman N. (2021). Associations of Sleep-disordered Breathing and Insomnia with Incident Hypertension and Diabetes. The Hispanic Community Health Study/Study of Latinos. Am. J. Respir. Crit. Care Med..

[33] Pinilla L., Santamaria-Martos F., Benítez I.D., Zapater A., Targa A., Mediano O., Masa J.F., Masdeu M.J., Minguez O., Aguilà M. (2021). Association of Obstructive Sleep Apnea with the Aging Process. Ann. Am. Thorac. Soc..

[34] Villalaín-Rodes I., García-Sánchez A., Durán M.A., García-Río F., Martínez J.G., Montejano-Milner R. (2025). Effect of Continuous Positive Airway Pressure Treatment on the Arteriole-to-Venule Ratio in Patients with Nonproliferative Diabetic Retinopathy and Obstructive Sleep Apnea: A Randomized Trial. Ophthalmic Surg. Lasers Imaging Retin..

[35] Moawd S.A., Azab A.R., Alrawaili S.M., Abdelbasset W.K. (2020). Inspiratory Muscle Training in Obstructive Sleep Apnea Associating Diabetic Peripheral Neuropathy: A Randomized Control Study. BioMed Res. Int..

[36] Zhao X., Yu X., Xin S., Zhang W., Zhang X., Ji L. (2021). Correlation between OSAHS and Early Peripheral Atherosclerosis Indices in Patients with Type 2 Diabetes Mellitus in China: A Cross-Sectional Inpatient Study. J. Diabetes Res..

[37] Bakker J.P., Baltzis D., Tecilazich F., Chan R.H., Manning W.J., Neilan T.G., Wallace M.L., Hudson M., Malhotra A., Patel S.R. (2020). The Effect of Continuous Positive Airway Pressure on Vascular Function and Cardiac Structure in Diabetes and Sleep Apnea. A Randomized Controlled Trial. Ann. Am. Thorac. Soc..

[38] Banghøj A.M., Krogager C., Kristensen P.L., Hansen K.W., Laugesen E., Fleischer J., Lebech Cichosz S., Poulsen P.L., Glymer Kirkegaard M., Thorsteinsson B. (2020). Effect of 12-week continuous positive airway pressure therapy on glucose levels assessed by continuous glucose monitoring in people with type 2 diabetes and obstructive sleep apnoea; a randomized controlled trial. Endocrinol. Diabetes Metab..

[39] Loffler K.A., Heeley E., Freed R., Meng R., Bittencourt L.R., Gonzaga Carvalho C.C., Chen R., Hlavac M., Liu Z., Lorenzi-Filho G. (2020). Continuous Positive Airway Pressure Treatment, Glycemia, and Diabetes Risk in Obstructive Sleep Apnea and Comorbid Cardiovascular Disease. Diabetes Care.

[40] Krogager C., Banghøj A.M., Poulsen P.L., Kirkegaard M.G., Thorsteinsson B., Tarnow L., Hansen K.W., Laugesen E. (2020). Effect of 12 weeks continuous positive airway pressure on day and night arterial stiffness and blood pressure in patients with type 2 diabetes and obstructive sleep apnea: A randomized controlled trial. J. Sleep Res..

[41] Neeland I.J., Eliasson B., Kasai T., Marx N., Zinman B., Inzucchi S.E., Wanner C., Zwiener I., Wojeck B.S., Yaggi H.K. (2020). The Impact of Empagliflozin on Obstructive Sleep Apnea and Cardiovascular and Renal Outcomes: An Exploratory Analysis of the EMPA-REG OUTCOME Trial. Diabetes Care.

[42] Jeon B., Sereika S.M., Callan J.A., Luyster F.S., DiNardo M.M., Chasens E.R. (2020). Age-Related Differences in Mood, Diabetes-Related Distress, and Functional Outcomes in Adults with Type 2 Diabetes Mellitus and Comorbid Obstructive Sleep Apnea and Insomnia. Diabetes Educ..

[43] Pavlou V., Lin S., Cienfuegos S., Ezpeleta M., Runchey M.C., Corapi S., Gabel K., Kalam F., Alexandria S.J., Vidmar A.P. (2024). Effect of Time-Restricted Eating on Sleep in Type 2 Diabetes. Nutrients.

[44] Vichova T., Petras M., Waldauf P., Westlake K., Vimmerova-Lattova Z., Polak J. (2025). Sleep-disordered breathing increases mortality in patients with diabetes. J. Clin. Sleep Med..

[45] Clements F., Vedam H., Chung Y., Smoleniec J., Sullivan C., Shanmugalingam R., Hennessy A., Makris A. (2025). Effect of Continuous Positive Airway Pressure or Positional Therapy Compared to Control for Treatment of Obstructive Sleep Apnea on the Development of Gestational Diabetes Mellitus in Pregnancy: Protocol for Feasibility Randomized Controlled Trial. JMIR Res. Protoc..

[46] Fang M., Wang D., Rooney M.R., Echouffo-Tcheugui J.B., Coresh J., Aurora R.N., Punjabi N.M., Selvin E. (2023). Performance of the Glucose Management Indicator (GMI) in Type 2 Diabetes. Clin. Chem..

[47] Barreto P.R., Diniz D.L.O., Lopes J.P., Barroso M.C., Daniele T.M.D.C., de Bruin P.F.C., de Bruin V.M.S. (2020). Obstructive Sleep Apnea and Wake-up Stroke—A 12 Months Prospective Longitudinal Study. J. Stroke Cerebrovasc. Dis..

[48] Rooney M.R., Aurora R.N., Wang D., Selvin E., Punjabi N.M. (2021). Rationale and design of the Hyperglycemic Profiles in Obstructive Sleep Apnea (HYPNOS) trial. Contemp. Clin. Trials.

[49] Rhodes S., Waters D., Brockway B., Skinner M. (2020). Physical activity behaviour and barriers to activity in adults at high risk of obstructive sleep apnoea. J. Prim. Health Care.

[50] Thaher O., Wollenhaupt F., Croner R.S., Hukauf M., Stroh C. (2024). Evaluation of the effect of sleeve gastrectomy versus Roux-en-Y gastric bypass in patients with morbid obesity: Multicenter comparative study. Langenbeck’s Arch. Surg..

[51] Pamidi S., Chapotot F., Wroblewski K., Whitmore H., Polonsky T., Tasali E. (2020). Optimal Continuous Positive Airway Pressure Treatment of Obstructive Sleep Apnea Reduces Daytime Resting Heart Rate in Prediabetes: A Randomized Controlled Study. J. Am. Heart Assoc..

